# H-type Tracheoesophageal Fistula in a Newborn: Determining the Exact Position of Fistula by Intra-operative Guidewire Placement 

**Published:** 2014-07-10

**Authors:** Anko Antabak, Tomislav Luetic, Drago Caleta, Ivan Romic

**Affiliations:** 1 Department of Surgery (Pediatric Surgery), University Hospital Centre, Zagreb; 2 Department of Pediatrics, University Hospital Centre, Zagreb

**Keywords:** Tracheoesophageal fistula, H-type fistula, Bronchoscopy, Guidewire

## Abstract

H-type tracheoesophageal fistula is a rare congenital anomaly that is seldom diagnosed in the neonatal age. Documenting it and then locating it at surgery are both difficult. A case is presented to highlight the diagnostic and therapeutic utility of trans-fistula guidewire placement.

## INTRODUCTION

H-type tracheoesophageal fistula (H-TEF) is a rare, life-threatening congenital anomaly, which accounts for 4- 5% of all esophageal atresias/ tracheoesophageal fistula (EA/TEF).[1,2] The clinical features are variable, but the commonest are recurrent respiratory symptoms, aspiration with cyanosis during feeding, and abdominal distension. All of this can lead to rapid respiratory failure, so early and well-planned management is necessary to prevent serious consequences. Because of the difficulty in identification of the level of the fistula even with modern diagnostics tests, preoperative or intraoperative bronchoscopic guidewire trans-fistula placement has been recommended nowadays.[3-5] We present here our experience with the same technique. 

## CASE REPORT

A full-term good weight female neonate presented to us on the 3rd day of life with cyanosis, dyspnea and tachypnea. The chest X-ray showed right lower lobe infiltrate and atelectasis, so the diagnosis of EA/TEF and aspiration pneumonia was made. The laboratory findings indicated leukocytosis (12,500/mm3) and increased levels of C-reactive protein (9,898 mg/l). Intravenous fluids, parenteral nutrition, and antibiotics were administered. Gastrografin esophogram demonstrated an isolated H-type TEF localized on the right postero-lateral side of the trachea (Fig. 1). The accurate level of the fistula could not be clearly evaluated. Bronchoscopy revealed a fistula between the posterior wall of the trachea and esophagus approximately half-way between the vocal cords and the carina of the trachea. The neonate was taken up for surgery on the 29th day of life. To decide whether to explore TEF through the cervical or thoracic route, we needed to know the exact location of TEF preoperatively. Following orotracheal intubation, trans-oral flexible bronchoscopy was performed, and the tracheal fistula opening was visualized. The vascular guidewire was carefully passed through the fistula opening into the esophagus. The bronchoscope was brought out, and the guidewire's position was confirmed by radiographs (Fig. 2). The fistula once identified at T2 level, we decided to use the lateral left cervical approach. Identification of the fistula intraoperatively was facilitated by traction on the wire by the anesthesiologist. Once the fistula was well exposed and isolated, it was doubly ligated and divided. The baby had an uneventful early postoperative outcome and is doing well on follow up over a year now.

**Figure:**
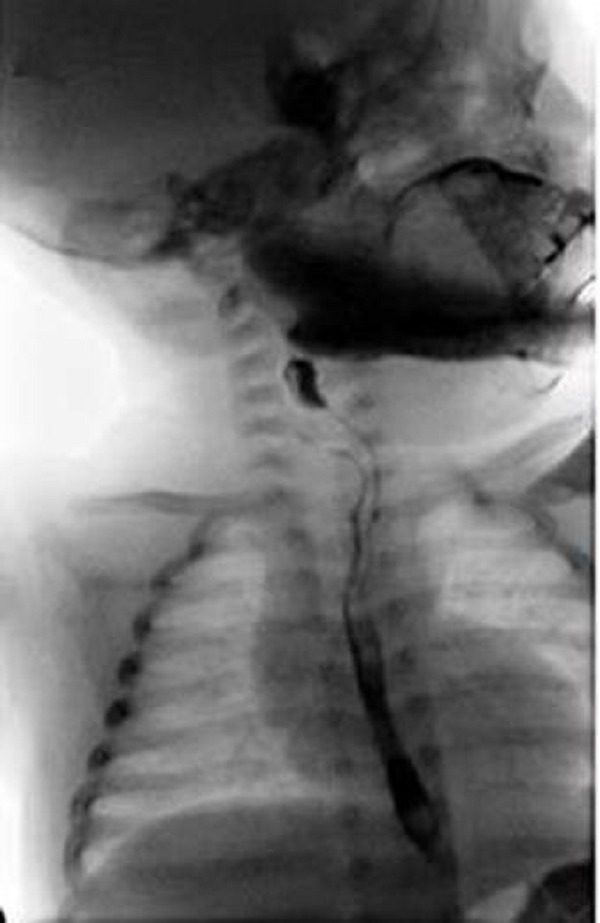
Figure 1: Tube esophagogram with gastrografin showing the contrast between esophagus and trachea indicating „H“-type esophageal fistula

**Figure:**
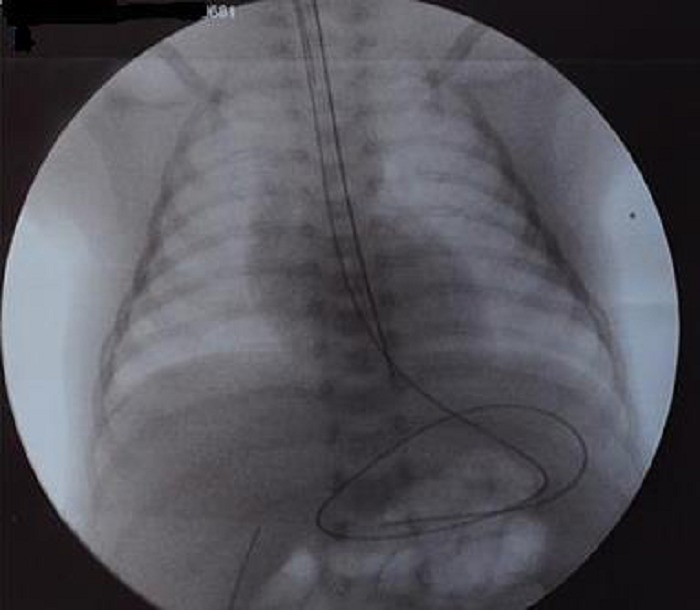
Figure 2: Chest radiograph showing the vascular guidwire which is entering the trachea, then passing through the tracheoesophageal fistula into the esophagus, and ending up curved in the stomach.

## DISCUSSION

H-TEF is a rare congenital anomaly with an incidence of about one in 100,000 live births. In view of such low incidence, there are not many reported cases or much scientific literature about the treatment, diagnosis and prognosis of this anomaly. Mortality rate for H-TEFs has significantly decreased during the last twenty years, mostly due to modern surgical techniques, diagnostic methods, and advanced postoperative treatment. However, unlike EA/TEF, the early diagnosis of H-TEF is sometimes difficult and delayed, so some cases may remain undiagnosed until late in infancy and even adulthood.[6] 

Both surgical and endoscopic management of the conditon have been described. Endoscopic management using fibrin occlusion, sclerosation, electrocautery, laser coagulation has a lower morbidity and mortality rate compared to the surgical approach,[7] but is associated with high recurrence rate. So, the surgery remains the mainstay treatment of the H-TEFs. For surgical correction, any of the three approaches could be used: lateral cervical, anterior cervical, and transthoracic approaches.[8] if the fistula is located above the level of T2 (which is the case in 70% of H-TEFs), a cervical approach is recommended. 

To know the exact location of H-TEF, radiological as well as endoscopic procedures have been described. Many surgeons nowadays recommend preoperative or intraoperative bronchoscopic guidewire trans-fistula placement.[3-5] This strategy has several advantages. Firstly, such a wire can be passed through a flexible bronchoscope, and it can be identified on a chest roentgenogram so we can verify the exact location of the fistula. Secondly, the wire can be palpated intraoperatively, which facilitates and simplifies identification of the fistula and preservation of surrounding structures. Instead of the vasuclar guidewire, Fogarty or ureteral catheters can be used.[3,8]

## Footnotes

**Source of Support:** Nil

**Conflict of Interest:** None

